# Short-term discontinuation of vagal nerve stimulation alters ^18^F-FDG blood pool activity: an exploratory interventional study in epilepsy patients

**DOI:** 10.1186/s13550-019-0567-9

**Published:** 2019-11-27

**Authors:** Ellen Boswijk, Renee Franssen, Guy H. E. J. Vijgen, Roel Wierts, Jochem A. J. van der Pol, Alma M. A. Mingels, Erwin M. J. Cornips, Marian H. J. M. Majoie, Wouter D. van Marken Lichtenbelt, Felix M. Mottaghy, Joachim E. Wildberger, Jan Bucerius

**Affiliations:** 1 Department of Radiology and Nuclear Medicine, Maastricht University Medical Center (MUMC+), P. Debyelaan 25, 6229 HX Maastricht, The Netherlands; 2 Cardiovascular Research Institute Maastricht (CARIM), Maastricht University, Universiteitssingel 50, 6229 ER Maastricht, The Netherlands; 3 School of Nutrition and Translational Research in Metabolism (NUTRIM), Maastricht University, Universiteitssingel 40, 6229 ER Maastricht, The Netherlands; 4 Department of Surgery, Erasmus Medical Center (EMC), Postbus 2040, 3000 CA Rotterdam, The Netherlands; 5 Department of Clinical Chemistry, Maastricht University Medical Center (MUMC+), P. Debyelaan 25, 6229 HX Maastricht, The Netherlands; 6 Department of Neurosurgery, Maastricht University Medical Center (MUMC+), P. Debyelaan 25, 6229 HX Maastricht, The Netherlands; 7 Department of Research & Development, Epilepsy Center Kempenhaeghe, Sterkselseweg 65, 5591 VE Heeze, The Netherlands; 8 Department of Neurology, Academic Center for Epileptology, Epilepsy Center Kempenhaeghe & Maastricht University Medical Center (MUMC+), P. Debyelaan 25, 6229 HX Maastricht, The Netherlands; 9 MHENS School of Mental Health & Neuroscience, Maastricht University, Universiteitssingel 40, 6229 ER Maastricht, The Netherlands; 10 School of Health Professions Education, Faculty of Health, Medicine and Life Sciences, Maastricht University, Universiteitssingel 60, 6229 ER Maastricht, The Netherlands; 11 Department of Nuclear Medicine, University Hospital RWTH Aachen, Pauwelsstraße 30, 52074 Aachen, Germany; 12 Department of Nuclear Medicine, Georg-August University Göttingen, Robert-Koch-Strasse 40, 37075 Göttingen, Germany

**Keywords:** Vagal nerve stimulation (VNS), Inflammation, Atherosclerosis, Positron emission tomography (PET), Metabolism

## Abstract

**Background:**

Vagus nerve activation impacts inflammation. Therefore, we hypothesized that vagal nerve stimulation (VNS) influenced arterial wall inflammation as measured by ^18^F-FDG uptake.

**Results:**

Ten patients with left-sided VNS for refractory epilepsy were studied during stimulation (VNS-on) and in the hours after stimulation was switched off (VNS-off). In nine patients, ^18^F-FDG uptake was measured in the right carotid artery, aorta, bone marrow, spleen, and adipose tissue. Target-to-background ratios (TBRs) were calculated to normalize the respective standardized uptake values (SUVs) for venous blood pool activity. Median values are shown with interquartile range and compared using the Wilcoxon signed-rank test. Arterial SUVs tended to be higher during VNS-off than VNS-on [SUV_max_ all vessels 1.8 (1.5–2.2) vs. 1.7 (1.2–2.0), *p* = 0.051]. However, a larger difference was found for the venous blood pool at this time point, reaching statistical significance in the vena cava superior [_mean_SUV_mean_ 1.3 (1.1–1.4) vs. 1.0 (0.8–1.1); *p* = 0.011], resulting in non-significant lower arterial TBRs during VNS-off than VNS-on. Differences in the remaining tissues were not significant. Insulin levels increased after VNS was switched off [55.0 pmol/L (45.9–96.8) vs. 48.1 pmol/L (36.9–61.8); *p* = 0.047]. The concurrent increase in glucose levels was not statistically significant [4.8 mmol/L (4.7–5.3) vs. 4.6 mmol/L (4.5–5.2); *p* = 0.075].

**Conclusions:**

Short-term discontinuation of VNS did not show a consistent change in arterial wall ^18^F-FDG-uptake. However, VNS did alter insulin and ^18^F-FDG blood levels, possibly as a result of sympathetic activation.

## Background

The vagus nerve (VN) is the longest cranial nerve and stretches from the medulla to the visceral organs. The nerve is comprised of multiple different nerve fibers, from afferent fibers from visceral organs (60–80% of VN fibers) to cholinergic parasympathetic pre-ganglionic efferent fibers using acetylcholine (ACh) as neurotransmitter [1]. Additionally, sympathetic nerve fibers join the vagus nerve from the cervical level downwards [2]. From as early as the end of the nineteenth century, effects of vagal nerve stimulation (VNS) have been studied in multiple conditions [3, 4]. Currently, VNS is approved as a therapeutic option in refractory epilepsy, reducing seizure frequency and severity, and refractory depression [3, 4]. The exact mechanism of VNS in these diseases is still unknown, but its effects are probably the result of central neuromodulatory mechanisms [4].

Current studies focus on new applications of VNS in a broad range of diseases including inflammatory conditions [4]. This indication is based on the effect of both the afferent and efferent VN as part of the so-called inflammatory reflex [5]. The afferent VN confers signals to the brain from inflammatory processes and activates the sympathetic nervous system and hypothalamic–pituitary–adrenal (HPA) axis, which consecutively inhibit chronic inflammation [6, 7]. The efferent VN is part of the cholinergic anti-inflammatory pathway (CAP) [8]. This is a complex feedback mechanism responsible for a central inhibition of peripheral inflammatory responses [9]. The exact mechanism of the CAP remains to be elucidated, but animal research has revealed the following: (1) stimulation of central, but not peripheral muscarinic acetylcholine receptors (mAChRs) results in an anti-inflammatory reaction [10]; (2) peripheral stimulation of α7-nicotinic acetylcholine receptors (nAChRs) expressed by macrophages inhibits TNF-α production [11]; (3) splenectomy disables the anti-inflammatory effect of VNS [12]; (4) surgical sympathetic denervation of the spleen and/or noradrenaline depletion prevents CAP function [13]; and (5) ACh-producing T cells are necessary for a functioning CAP [14]. These mechanisms may also be involved in the inflammatory cascade of the atherosclerotic disease process [15].

In this exploratory subgroup analysis, we aimed to study the anti-inflammatory effect of VNS on the arterial wall by comparing arterial wall fluorodeoxyglucose (^18^F-FDG) uptake on positron emission tomography computed tomography (PET/CT) during VNS and during a period in which VNS was switched off within the same patient. We expected VNS to reduce arterial inflammation and thus ^18^F-FDG uptake, since arterial wall ^18^F-FDG uptake as visualized and measured with PET/CT, is an established marker for inflammation in atherosclerosis [16]. However, ^18^F-FDG is a glucose analogue, and thus behaves as glucose and is affected by VNS’ interference with glucose metabolism.

## Methods

### Study protocol approval and study aim

This is a subgroup analysis of a prospective trial, which included a total of 15 patients and studied the effects of VNS on the activation of brown adipose tissue with ^18^F-FDG PET/CT. The initial study was approved by the local medical ethics committee of the academic hospital and the University of Maastricht (AZM/UM) and registered in the clinical trial register at ClinicalTrials.gov under NCT01491282 [17]. All participants gave written informed consent before the onset of the study. Participants were selected from patients with refractory epilepsy treated with VNS who visited the outpatient clinic of our tertiary expertise center for epilepsy. Details on in- and exclusion criteria and VNS principles can be found in Table 1 and in the original study by Vijgen et al. [17]. This subgroup analysis aimed to include the ten participants from the initial study population, who underwent ^18^F-FDG PET/CT once with active (VNS-on) and once with deactivated VNS (VNS-off) to study the effect of VNS on arterial wall inflammation. For this additional analysis, the local medical ethics committee waived additional informed consent.

**Table 1:** In- and exclusion criteria

Inclusion	Exclusion
• 18-65 years of age	• Daily seizures
• VNS for refractory epilepsy	• Pregnancy
• Stable VNS and epilepsy medication > 1 month	• Ketogenic diet
	• Mental retardation
	• Psychological instability

### Subject characteristics

The ten patients were six females and four males with a median age of 45 years (interquartile range (IQR) 33–52). Stimulation parameters can be found in Table 2. Characteristics for all subjects (*n* = 10) can be found in Table 3. At the time of the first (VNS-on) scan, the median time period since VNS implantation had been 56 months (IQR 46–66).

**Table 2:** Stimulation parameters

Subject	Output current (mA)	Frequency (Hz)	Pulse width (μs)	On-period (s)	Off-period (s)
01	2.25	30	250	30	300
02	0.75	30	250	30	300
03*	1.5	30	250	7	20
04	0.75	30	250	30	300
06	1.25	30	500	30	300
10	2	30	250	30	300
11	1	30	250	30	300
12	1.75	30	250	7	18
13	2.25	30	250	30	300
14	2	30	250	30	300

In subject 03 (*), the VNS-off scan could not be reconstructed

**Table 3:** Subject characteristics (*n* = 10)

Age (years)	45 (33–52)
Sex (number of males)	4
Weight (kg)	67.4 (58.1–75.8)
BMI (kg/m^2^)	24.4 (22.6–26.2)
Time since implantation of VNS (months)	56 (46–66)
Time between experiments (days)	14 (14–49)

Median values are given with the IQR between brackets. Abbreviations: *BMI* body mass index

### Study design

Ten participants underwent ^18^F-FDG PET/CT under thermoneutral and fasted conditions twice; once with active VNS, and once after deactivation of VNS. The median time period between the two scans was 14 (IQR 14–49) days. Because the VNS-off scan of one participant could not be adequately reconstructed, this patient was excluded from the PET-data analysis, resulting in the nine participants included in this analysis. However, all other data from this patient were complete and thus used for analyses of the non-PET-related data.

During deactivation, VNS was turned off in the morning at 9.30 am and turned back on at the end of the test day around 2 pm. All PET/CTs were performed at 1 pm, 3.5 h after VNS was switched off. On the same day, participants also underwent additional tests to study, among other things, energy expenditure. This was done by indirect calorimetry using a ventilated hood system (Omnical, Maastricht, the Netherlands). Details about the techniques used can be found in the original study by Vijgen et al. [17].

E.B. had full access to all data and takes responsibility for data integrity and analysis.

### Laboratory tests

On the days of either scan, fasting blood (EDTA plasma and serum) was drawn right before the measurements were done. On the day of the VNS-off scan, blood samples were taken after VNS was switched off, right before injection of the tracer. Glucose was measured using a hexokinase-based assay (Cobas 6000, Roche Diagnostics, Mannheim, Germany) with a reference range of 3.1–7.8 mmol/L. Insulin was measured with Immulite XPi, Siemens Healthcare, Erlangen, Germany). Initial results were in mU/L and were converted to SI units with a factor of 6.00 (1 mU/L = 6.00 pmol/L) [18] with a reference range between 12 and 150 pmol/L. C-reactive protein (CRP) was measured using a turbidimetric assay (Cobas 8000) with a reference range of < 10 mg/L and a detection limit of > 1 mg/L.

### PET protocol

A mean standard activity of 75 MBq ^18^F-FDG was injected 1 h before scanning on a Gemini TF PET/CT system (Philips Healthcare, Best, The Netherlands). For attenuation correction and anatomical co-localization of the PET signal, a low-dose native whole-body CT protocol (120 kVp, 30 mAs) was used. All scans were performed after an overnight fast and confirmation of appropriate glucose levels (< 7 mmol/L).

### Image analysis

Analyses of the PET data were carried out using a commercially available, dedicated workstation (Extended Brilliance Workspace V4.5.3.40140; Philips Healthcare, Best, The Netherlands). Circular regions of interest (ROIs) were placed to delineate the outer contour of the vessel wall of the common carotid artery and the aorta (ascending aorta, aortic arch, thoracic descending aorta (until the diaphragm) and abdominal aorta (until the iliac bifurcation or as low as sufficiently imaged)). Since all participants had a VNS implanted close to the left carotid sinus, only the right carotid artery was included in the analysis in order to exclude local effects of the device on ^18^F-FDG uptake in the left carotid artery. Standard circular ROIs (diameter 20 mm) were placed on the center of each thoracic and lumbar vertebra to measure bone marrow (BM) activity. For the spleen, standard circular ROIs (diameter 20 mm) were placed centrally in the spleen on each transversal plane. Visceral adipose tissue (VAT) was measured with three circular 10 mm ROIs placed in intra-peritoneal fat at the level of the umbilicus. ROIs of the same size were placed in the nuchal subcutaneous adipose tissue (SAT) and in SAT at the level of the xiphoid. The ^18^F-FDG activity within each ROI was measured as the mean and maximum standardized uptake value (SUV_mean_ and SUV_max_, respectively). SUV represents activity concentration per ROI normalized for the administered activity, corrected for decay (dependent on injected activity and time) and body weight of the subject. The SUV_mean_ and SUV_max_ of all ROIs were normalized for blood pool activity by calculating target-to-background ratios (TBRs). To achieve this, arterial SUVs were divided by the averaged SUV_mean_ (_mean_SUV_mean_) of at least three standard circular ROIs (diameter 4 mm) placed in the lumen of a reference vein. In the case of the carotid artery and nuchal SAT TBRs, the jugular vein (JV) was used as the reference vein. For all other TBRs, the _mean_SUV_mean_ of the vena cava superior (VCS) was used to calculate the TBR. Blood pool normalization of the SUV_mean_ and SUV_max_ resulted in the corresponding TBR_mean_ and TBR_max_. Average TBRs were calculated per vascular territory. ROIs were excluded from further analysis in case spill-in of activity from adjacent structures was suspected or artifacts prevented the drawing of accurate ROIs.

### Statistical analysis

Because of the small sample size, continuous data are presented with their median and interquartile range (Q1–Q3) and compared using the non-parametric Wilcoxon signed rank test. Correlation was tested using the Spearman correlation coefficient. Statistical significance was defined at the 95% confidence level (*p* < 0.05). All statistical analyses were performed with IBM SPSS Statistics for Mac OS X, version 24 (2015).

## Results

### Changes in energy expenditure and laboratory tests

**Fig. 1:**
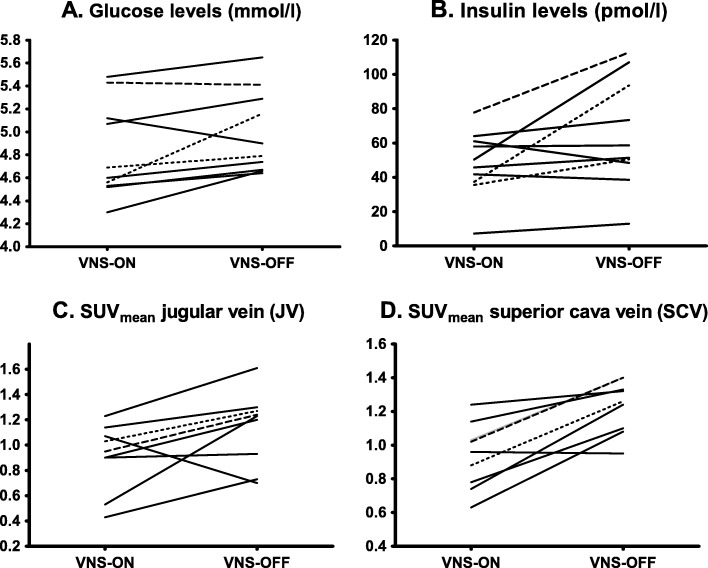
Change in glucose, insulin and venous ^18^F-FDG levels after VNS discontinuation. The change in glucose levels (**a**), insulin levels (**b**), and average SUV_mean_ values for the jugular veins (**c**) and vena cava superior (**d**) are shown per subject (*n* = 10 for **a** and **b** and *n* = 9 for **c** and **d**, respectively). Generally, these measurements increased in most patients when VNS was switched off (VNS-OFF) in comparison to functional VNS (VNS-ON). The dotted lines represent subject 03 and 12 in (**a**, **b**), whose stimulation parameters differed from those of the other subjects. Of these two subjects, subject 12 shows the steepest increase in glucose and insulin levels. In (**c**, **d**), the dotted line represents values of subject 12 because the VNS-OFF scan of subject 03 could not be used for analysis. The dashed line represents subject 14, who was the only subject with multiple cardiovascular risk factors

When VNS was switched off, energy expenditure (EE) decreased in nine out of ten patients (*p* = 0.047). In addition, glucose levels tended to be higher after VNS was switched off (4.8 mmol/L (4.7–5.3) vs. 4.6 mmol/L (4.5–5.2); Fig. 1a), although this was not statistically significant (*p* = 0.053). However, insulin levels increased significantly in the VNS-off state (55.0 pmol/L (45.9–96.8) vs. 48.1 pmol/L (36.9–61.8); *p* = 0.047; Fig. 1b). CRP-levels did not significantly differ between scans (1.0 mg/L (0.8–2.0) during VNS-off vs. 1.2 mg/L (1.0–2.7) during VNS-on; *p* = 0.445). Details can be found in Table 4. Also see Additional file 1: Figure S1 for individual CRP levels.

**Table 4:** Laboratory tests, energy expenditure, core temperature, ^18^F-FDG activity, and blood pool activity

			VNS-on	VNS-off	p-value
CRP (mg/L)		*N* = 10	1.2 (1.0-2.7)	1.0 (0.8–2.0)	0.445
Glucose (mmol/L)		*N* = 10	4.6 (4.5–5.2)	4.8 (4.7–5.3)	0.053
Insulin (pmol/L)		*N* = 10	48.1 (36.9–61.8)	55.0 (45.9–96.8)	**0**.**047**
Energy expenditure (J/s)		*N* = 10	70.4 (61.1–75.8)	68.4 (59.7–74.3)	**0**.**047**
^18^F-FDG activity (MBq)		*N* = 9	76 (74–82)	79 (73–81)	0.766
Blood pool activity	_mean_SUV_mean_ JV	*N* = 9	0.9 (0.7–1.1)	1.2 (0.8–1.3)	0.066
	_mean_SUV_mean_ VCS	*N* = 9	1.0 (0.8–1.1)	1.3 (1.1–1.4)	**0**.**011**

Median values are given with the IQR between brackets. *P* values in bold indicate statistically significant findings. Abbreviations: *CRP* C-reactive protein, *SUV* standardized uptake value, *JV* jugular vein, *SCVCS* vena superior cava veinsuperior

Administered ^18^F-FDG activity did not differ between scans (see Table 4). ^18^F-FDG blood pool activity increased when VNS was turned off (SUV_mean_ 1.2 (0.8–1.3) vs. 0.9 (0.7–1.1), *p* = 0.066 for the jugular vein (Fig. 1c) and 1.3 (1.1–1.4) vs. 1.0 (0.8–1.1), *p* = 0.011 for the VCS (Fig. 1d); see also Table 4). Blood pool activity did not correlate to glucose or insulin levels when data of both timepoints were combined. Only the off-scan insulin levels correlated to the corresponding _mean_SUV_mean_ of the VCS (Spearman ρ = 0.745; *p* = 0.021). Furthermore, the blood pool activity, glucose, or insulin levels did not correlate to EE, nor did the difference in blood pool activity correlate to the difference in EE.

### PET/CT image quality

In one of the VNS-on scans, ROIs could not be placed accurately around the lumen of the abdominal aorta as well as in the spleen and xiphoid SAT, due to movement of the participant between the PET and CT scan, which resulted in a disturbed attenuation and scatter correction. In another VNS-on scan, VAT could not be analyzed due to a truncation artifact because of high activity outside the field of view of the accompanying low-dose CT reconstruction. In one scan of one participant, the right carotid artery could not be properly delineated and in another participant, spill-in of intestinal activity was suspected in the VAT ROIs. Additionally, in the case of three lean patients, the amount of adipose tissue was insufficient to ensure adequate ROI placement in one or more of the adipose tissue compartments. In total, the analysis was based on the following number of participants; carotid artery *n* = 8; abdominal aorta *n* = 8; spleen *n* = 8; nuchal SAT *n* = 7; xiphoid SAT *n* = 5; VAT *n* = 6. All other PET/CT analyses were based on nine participants.

### Effect of VNS on ^18^F-FDG activity in the arterial wall, hematopoietic organs, and adipose tissue

Arterial SUVs tended to be higher after VNS was turned off. However, this difference was only significant in the aortic arch (*p* = 0.012) and the thoracic aorta (*p* = 0.038). In contrast, arterial TBRs tended to be non-significantly lower after VNS was switched off than when VNS was still turned on. For average arterial SUV and TBR values per subject, see Additional file 2: Figure S2.

In line with the findings in the arterial territories, SUVs tended to be higher in the spleen, BM, and SAT after VNS was turned off, albeit not statistically significant. Only in VAT, a slight though non-significant decrease in SUVs could be observed after VNS was turned off. Furthermore, TBRs of the spleen, BM, SAT, and VAT tended to increase after VNS was turned off compared to the on-mode values. None of these differences reached statistical significance. For details, see Table 5.

**Table 5:** Maximum standardized uptake values (SUV_max_) and target-to-background ratios (TBR_max_)

		VNS-on	VNS-off	*p* value
Right carotid artery^a^	SUV_max_	1.3 (0.9–1.6)	1.5 (1.2–1.8)	0.093
	TBR_max_	1.5 (1.2–1.7)	1.2 (1.2–1.7)	0.484
Ascending aorta	SUV_max_	1.8 (1.3–2.0)	1.9 (1.7–2.4)	0.066
	TBR_max_	1.8 (1.6–2.0)	1.6 (1.5–1.7)	0.139
Aortic arch	SUV_max_	1.7 (1.2–2.1)	1.9 (1.5–2.4)	**0**.**012**
	TBR_max_	1.6 (1.5–2.1)	1.5 (1.4–1.8)	0.086
Thoracic aorta	SUV_max_	1.7 (1.2–2.1)	1.9 (1.6–2.4)	**0**.**038**
	TBR_max_	1.7 (1.5-2.1)	1.5 (1.4-1.7)	0.139
Abdominal aorta^a^	SUV_max_	1.7 (1.1-1.9)	1.7 (1.5-2.0)	0.401
	TBR_max_	1.6 (1.5–1.9)	1.4 (1.4–1.5)	0.093
All vessels	SUV_max_	1.7 (1.2–2.0)	1.8 (1.5–2.2)	0.051
	TBR_max_	1.7 (1.5–1.9)	1.5 (1.4–1.6)	0.086
Spleen^a^	SUV_max_	1.6 (1.1–2.0)	1.8 (1.7–2.1)	0.069
	TBR_max_	1.7 (1.4–2.0)	1.5 (1.4–1.6)	0.327
Bone marrow	SUV_max_	2.4 (1.4–2.6)	2.3 (2.0–2.6)	0.594
	TBR_max_	2.1 (1.7–2.5)	1.8 (1.7–1.9)	0.110
Nuchal SAT^b^	SUV_max_	0.4 (0.3–0.5)	0.5 (0.5–0.6)	0.310
	TBR_max_	0.4 (0.4–0.6)	0.4 (0.4–0.5)	0.499
Xiphoid SAT^c^	SUV_max_	0.5 (0.4–0.8)	0.6 (0.5–0.9)	0.273
	TBR_max_	0.6 (0.4–0.8)	0.4 (0.3–0.6)	0.225
VAT^d^	SUV_max_	0.7 (0.5–0.8)	0.6 (0.5–0.7)	0.462
	TBR_max_	0.7 (0.5–0.8)	0.4 (0.3–0.6)	0.173

When not stated otherwise, median values for the nine patients are given with the IQR between brackets. ^a^*n* = 8 participants, ^b^*n* = 7 participants, ^c^*n* = 5 participants, ^d^*n* = 6 participants. *P* values in bold indicate statistically significant findings

### Correlations between TBR_max_ and laboratory tests

There were no statistically significant correlations between TBR_max_ values of the vessels or hematopoietic organs, and CRP, glucose, or insulin levels. Only the off-scan insulin levels correlated to the corresponding TBR_max_ of xyfoid SAT (spearman ρ = − 0.829; *p* = 0.042).

## Discussion

In this exploratory analysis, we aimed to investigate a difference in arterial wall ^18^F-FDG uptake as a result of a change in inflammatory status when chronic VNS was discontinued. Switching off VNS only changed the SUV_max_ significantly in some arterial territories. There was no significant change in arterial TBR_max_. However, even though VNS was only discontinued for 3.5 h, insulin levels and ^18^F-FDG blood pool activity did increase. The latter resulted in an unexpected non-significant decrease in arterial TBRs. It appears that VNS alters ^18^F-FDG (and thus probably also glucose) distribution throughout the body.

Previous research has shown arterial wall ^18^F-FDG uptake to correlate to both plaque macrophage content and the risk of future cardiovascular events [19, 20]. TBR is an accepted and commonly used outcome measure to study arterial wall inflammation [16]. The relatively small size of the arterial wall in comparison to the spatial resolution of ^18^F-FDG PET/CT makes delineating the vascular wall challenging and in the current method, the lumen is therefore included in the ROI. TBR is the accepted method to compensate for this. The interpretation of this correction is of particular interest in this study, since SUV and TBR outcomes seem to contradict one another. When VNS was switched off, SUVs were higher than during VNS-on, suggesting an increase in arterial wall inflammation due to a decrease in the anti-inflammatory effect of VNS. This is in line with our hypothesis that VNS decreases arterial wall inflammation. However, the effect of VNS on blood pool activity was larger at this time point, which resulted in a non-significant decrease in TBRs after VNS was switched off. The contradictory findings of the TBRs with respect to the SUVs and to our hypothesis, and the relatively limited intervention, led us to explore alternative hypotheses to account for the changes in arterial wall ^18^F-FDG uptake beyond an effect on local inflammation.

Although ^18^F-FDG has proven its relevance in atherosclerotic inflammation imaging, it is not specific for this disease. As a glucose analogue, ^18^F-FDG’s uptake is influenced by the same factors influencing glucose uptake—demand and supply—and by competition with dietary glucose. First of all, glucose demand is increased in active tissues. For instance, an increased ^18^F-FDG uptake can be observed in tumors and inflammatory processes. Secondly, glucose supply is dependent on blood flow and distribution. Thirdly, ^18^F-FDG uptake in a specific tissue depends on its competition with dietary glucose and with the demand for glucose from other tissues. Because of this competition, patients should be fasted and have blood glucose levels lower than 7 mmol/L before an ^18^F-FDG PET/CT [16]. While our investigation aimed to image a change in inflammatory activity due to VNS treatment, it is possible that VNS also affected ^18^F-FDG distribution via blood flow and/or systemic glucose metabolism.

Multiple mechanisms can be proposed to explain an altered ^18^F-FDG distribution due to VNS. The VN includes multiple fiber types, both afferent and efferent, projecting to and originating from various nuclei in the brain to and from almost every visceral organ [1]. Most efferent fibers are cholinergic parasympathetic fibers, but the VN also interacts with the sympathetic nervous system on multiple levels [2]. The VNS electrode is positioned in such a way that pulses are primarily directed in the afferent direction. In addition, extrapolation from animal studies shows that, most likely, commonly used VNS output currents stimulate mainly somatic and visceral afferent A-fibers [1, 4]. Concurrently, “side-effects” of VNS are also most likely to result from afferent rather than efferent stimulation.

The afferent VN is suggested to activate the sympathetic nervous system in order to control inflammation [7, 21], cardiac output, and blood pressure [22]. Stimulation of the sympathetic nervous system could cause blood flow to be altered due to peripheral, renal, and intestinal vasoconstriction [23]. If this is indeed the case, one might expect an increase in blood pressure under VNS stimulation. However, several studies in rats appear to show no increase or even a decrease in blood pressure by VNS [24–28]. In VNS trials in humans, heart rate tends to be the main measure for cardiovascular safety while possible effects on blood pressure are less well studied [29]. Short-term (120 s) transcutaneous VNS did not show an effect on blood pressure in healthy volunteers [30], nor did VNS in epilepsy patients at 16 weeks after implantation [31]. Studies in epilepsy patients, which had VNS treatment for a longer period, did not show a difference in blood pressure between the on- and off-period of VNS stimulation [29, 32]. In addition, despite an increase in sympathetic responsiveness of blood pressure, one study showed long-term VNS to result in a decrease in blood pressure compared to baseline [32]. We therefore do not suspect altered blood flow to be the main cause for the observed effect on ^18^F-FDG uptake in the arterial wall and blood pool.

An alternative explanation for the observed ^18^F-FDG distribution due to a sympathetic effect of VNS is the increase of insulin-independent glucose uptake in peripheral tissues, mainly the skeletal muscles [33–35]. This could explain the lower blood pool activity under VNS, because of an increased uptake of ^18^F-FDG in the muscles, which were outside the field of view for the most part.

The VN also influences glucose metabolism through systemic glucose storage and release, and through glucagon and insulin levels. In the current study, both glucose and insulin levels tended to be higher when VNS was switched off than during VNS. This seems to be in agreement with findings of simultaneous afferent and efferent stimulation of the VNS in rats. Afferent VNS (both with and without concurrent efferent stimulation) for a time period of 120 min resulted in a strong and sustained increase in glucose levels, probably due to an increased glucose release from the liver combined with suppressed insulin secretion [36, 37]. Pure efferent VNS mainly resulted in increased insulin levels [36]. An exclusive increase in glucose levels could explain a lower uptake of ^18^F-FDG in the target tissues due to an enhanced competition with glucose. In theory, however, this would also increase the ^18^F-FDG blood pool activity. In addition, previous research has shown ^18^F-FDG competition with dietary glucose to only be relevant when glucose levels exceed 7 mmol/L [16]. A sole increase of insulin would increase general glucose- and thus ^18^F-FDG uptake and would result in a decrease of the blood pool activity of the tracer. Furthermore, since insulin increases glucose (and thus ^18^F-FDG) uptake in multiple tissues, an increase in insulin levels could also result in lower uptake in a specific tissue, due to an increased competition with other tissues. Excluding an effect of insulin-independent peripheral uptake, a proportional increase of insulin and glucose would cause the net result of ^18^F-FDG uptake to remain unchanged. It appears therefore that our results are best explained by a combination of afferent and efferent VN stimulation in which increased insulin levels, and possibly insulin-independent increased peripheral uptake, probably play a more significant role than the increased glucose levels.

It is important to realize that both the anatomy of the VN and VNS settings used in animal models differ from the human situation. In the abovementioned studies, animals were treated with continuous VNS, whereas in humans, stimulation is non-continuous. Indeed, a recent retrospective study showed an effect on blood glucose levels of chronic VNS to depend on stimulation parameters [38]. A long stimulation ON-period and a short stimulation OFF-period were associated with higher glucose levels on follow-up in epilepsy patients treated with VNS. The most commonly used stimulation parameters of our subjects (30 s ON, 300 s OFF) fall between the estimated “neutral effect parameters” of the abovementioned study and the stimulation parameters of the two subjects with divergent parameters (subject 03 and 12) fall within the “glucose lowering parameters” [38]. Although, subject 12 shows a relatively steep increase in glucose and insulin levels after VNS is switched off, there is no clear trend among the other subjects, and a larger study would be necessary to further investigate this hypothesis.

Although, we were unable to show an anti-inflammatory effect of VNS on atherosclerosis, probably due to the abovementioned effects on glucose metabolism, it should also be taken into account that our population of refractory epilepsy patients was relatively young and only one participant (subject 14) had known classic cardiovascular risk factors. Remarkably, this middle-aged male subject, who was overweight and had both hypertension and hypercholesterolemia, did show an increase in arterial TBRs after VNS was switched off in contrast to most participants (Additional file 2: Figure S2). Of course, no conclusions can be drawn from this, since it concerns a single patient, but it does strengthen our conviction that VNS might indeed offer a therapeutic option for atherosclerosis and other chronic inflammatory diseases.

### Strengths and limitations

A major strength of this study is that all patients served as their own control. Since the same patients, the same scan protocol, the same timing, and the same activity of ^18^F-FDG were used for both the VNS-on and VNS-off scans.

Important drawbacks of this study are the small sample size and the short duration that VNS was turned off. It was considered unethical to turn the VNS off for a longer time period. A longer VNS-off period might have resulted in larger differences in measurements in comparison to VNS-on, which would possibly have affected the statistical significance of our findings. In addition, we expect this to affect the concurring consequences of VNS, such as changes in blood glucose levels, to a similar extent. In addition, we compared long-term stimulation to short-term discontinuation, which is possibly quite different from a VNS-naïve situation.

Another limitation is the lack of basic physical tests, such as blood pressure and heart rate, which could have supported our hypothesis of a sympathetic effect of VNS.

It is also important to note that we did not perform partial volume correction, since the used PET/CT system did not feature a resolution recovery reconstruction algorithm. This might have resulted in an underestimation of the arterial wall uptake. However, since this is true for both the VNS-on and the VNS-off scans, we expect that this did not significantly affect our results.

In conclusion, short-term discontinuation of VNS altered SUV_max_ for ^18^F-FDG in some arterial territories, but not TBR_max_. However, this intervention affected the venous blood pool activity and insulin levels. It seems therefore that VNS might have an effect on glucose metabolism, possibly as a result of sympathetic activation.

## Supplementary information

**Additional file 1: Figure S1.** Change in C-reactive protein levels after VNS-discontinuation. Depicted are the C-reactive protein (CRP) levels for individual subjects (*n*=10). The dotted lines represent subject number 03 and 12, whose stimulation parameters differed from the other subjects. Subject 12 had the highest CRP value at the VNS-ON scan. The dashed line represents subject number 14, who was the only subject with cardiovascular risk factors. In contrast to most other subjects, CRP was higher in this subject after VNS was switched off than during the VNS-ON scan.

**Additional file 2: Figure S2.** Changes in average SUV_mean_, SUV_max_, TBR_mean_ and TBR_max_ for all measured arterial territories after VNS-discontinuation. Depicted are the average SUV_mean_, SUV_max_, TBR_mean_ and TBR_max_ for all measured arterial territories combined for each individual subject (*n*=9). These average values are based on those of the right carotid artery and of four areas in the aorta in *n*=7. In one subject the values of both time points are based on the four arterial territories excluding the abdominal aorta, because of a disturbed scatter and attenuation correction of the VNS-OFF scan. In another subject, the carotid artery is not included in the average values, because it could not be sufficiently delineated. The dotted line represents subject number 03, whose stimulation parameters differed from the other subjects. The dashed line represents subject number 14, who was the only subject with cardiovascular risk factors. In contrast to most other subjects, TBRs were higher in this subject after VNS was switched off.

## Data Availability

The datasets analyzed during the current study are not publicly available because of ethical considerations but are available from the corresponding author on reasonable request.

## Abbreviations

^18^F-FDG: ^18^F-fluorodeoxyglucose
ACh: Acetylcholine
BM: Bone marrow
CAP: Cholinergic anti-inflammatory pathway
CRP: C-reactive protein
CT: Computed tomography
EE: Energy expenditure
HPA axis: Hypothalamic–pituitary–adrenal axis
JV: Jugular vein
mAChR: Muscarinic ACh receptor
nAChR: Nicotinic ACh receptor
PET: Positron emission tomography
ROI: Region of interest
SAT: Subcutaneous adipose tissue
SUV: Standardized uptake value
VCS: Vena cava superior
TBR: Target-to-background ratio
VAT: Visceral adipose tissue
