# Supplementary figures and images for: Short-term discontinuation of vagal nerve stimulation alters 18F-FDG blood pool activity: an exploratory interventional study in epilepsy patients
Source: EJNMMI Res. 2019 Nov 27;9:101. doi: 10.1186/s13550-019-0567-9 (PMC6879675; doi:10.1186/s13550-019-0567-9)

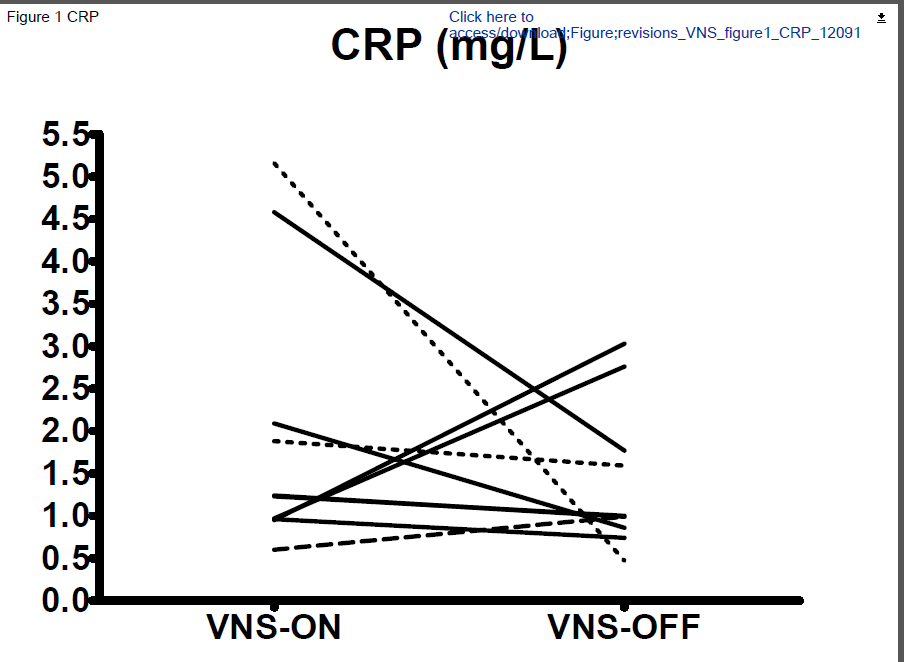

Supplement: Supplementary file 1 — Additional file 1: Figure S1. Change in C-reactive protein levels after VNS-discontinuation. Depicted are the C-reactive protein (CRP) levels for individual subjects (n=10). The dotted lines represent subject number 03 and 12, whose stimulation parameters differed from the other subjects. Subject 12 had the highest CRP value at the VNS-ON scan. The dashed line represents subject number 14, who was the only subject with cardiovascular risk factors. In contrast to most other subjects, CRP was higher in this subject after VNS was switched off than during the VNS-ON scan. [file 13550_2019_567_MOESM1_ESM.docx]

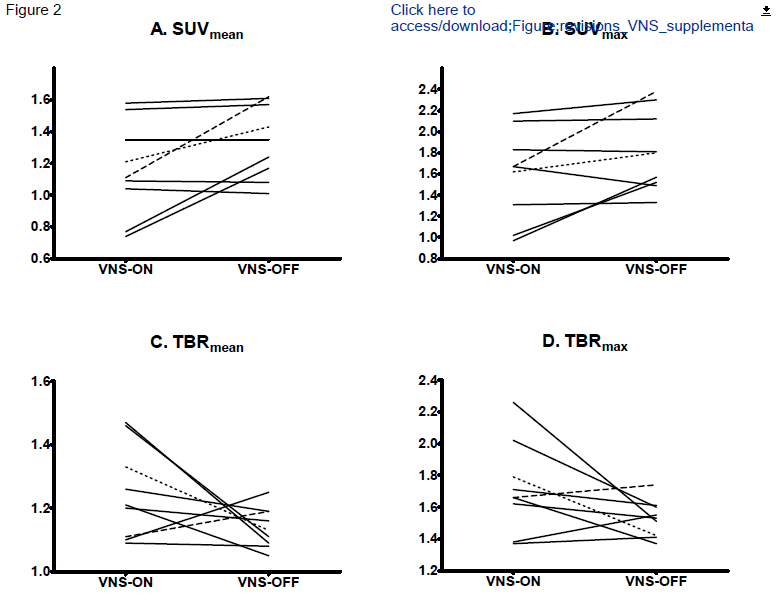

Supplement: Supplementary file 2 — Additional file 2: Figure S2. Changes in average SUVmean, SUVmax, TBRmean and TBRmax for all measured arterial territories after VNS-discontinuation. Depicted are the average SUVmean, SUVmax, TBRmean and TBRmax for all measured arterial territories combined for each individual subject (n=9). These average values are based on those of the right carotid artery and of four areas in the aorta in n=7. In one subject the values of both time points are based on the four arterial territories excluding the abdominal aorta, because of a disturbed scatter and attenuation correction of the VNS-OFF scan. In another subject, the carotid artery is not included in the average values, because it could not be sufficiently delineated. The dotted line represents subject number 03, whose stimulation parameters differed from the other subjects. The dashed line represents subject number 14, who was the only subject with cardiovascular risk factors. In contrast to most other subjects, TBRs were higher in this subject after VNS was switched off. [file 13550_2019_567_MOESM2_ESM.docx]
